# Computed tomographic heterogeneous enhancement of spleen in healthy cats: comparing with diffuse infiltrative splenic lesions

**DOI:** 10.3389/fvets.2024.1276984

**Published:** 2024-05-15

**Authors:** Yooyoung Lee, Dongwoo Chang, Sojin Kim, Miju Oh, Jiyoung Ban, Minju Lee, Jinhwa Chang, Jisoo Ahn, Taegeon An

**Affiliations:** ^1^Section of Veterinary Medical Imaging, Veterinary Teaching Hospital, College of Veterinary Medicine, Chungbuk National University, Cheongju, Republic of Korea; ^2^Korea Animal Medical Center, Cheongju, Republic of Korea; ^3^Daegu Animal Medical Center, Daegu, Republic of Korea; ^4^Lucid Animal Medical Center, Seoul, Republic of Korea

**Keywords:** cat, spleen, contrast-enhanced computed tomography, heterogeneous enhancement of the spleen, diffuse infiltrative splenic lesions

## Abstract

**Introduction:**

Contrast-enhanced computed tomography (CT) of the spleen in dogs and cats often displays a heterogeneous enhancement pattern. This study aimed to describe the CT appearances and duration of heterogeneous splenic enhancement in clinically healthy cats and to compare those enhancements with diffuse infiltrative splenic lesions (DISL).

**Methods:**

Spleens of 14 healthy cats were imaged using contrast-enhanced CT protocols which were obtained at 10, 25, and 45 s, and then every 40 s thereafter until 245 s had past from the initiation of contrast medium injection. The presence of transient splenic heterogeneity was evaluated. In addition, the relationships of certain variables including age, weight, systolic blood pressure, and splenic volume to the duration and the degree of splenic enhancement were determined. Also, medical records and CT images of five cats with DISL were retrospectively evaluated.

**Result:**

Transient heterogeneous enhancement of the spleen was observed in all 14 healthy cats, and the maximum heterogeneity was observed 25 s after the injection. Splenic heterogeneity lasted more than 5 min in nine of 14 cats (64.3%). No statistically significant relationships were seen between the duration and degree of splenic heterogeneity in the images taken 25 s after the injection and variables including weight, age, systolic blood pressure, and splenic volume.

**Discussion:**

Compared to the healthy group, early homogeneous splenic enhancement along with generalized splenomegaly was observed in all cats with DISL. Transient splenic heterogeneity is highly common in cats undergoing contrast-enhanced CT even in the generally scanned delayed phases, which can help with the interpretation of CT images of feline spleens. In addition, our results suggest that homogeneous splenic enhancement in post-contrast CT scans along with splenomegaly on CT images could be useful as a diagnostic indicator of DISL in cats.

## 1 Introduction

Heterogeneous enhancement of the spleen occurring during contrast-enhanced CT is a well-documented phenomenon in humans (1–5). The cause of these heterogeneous enhancement patterns is thought to be related to different vascular pathways and flow rates through the cords of red and white pulp, according to the unique anatomic structure of the spleen (1, 6, 7). These heterogeneous patterns of the spleen have been also seen in dogs and cats, as helical CT with rapid speed and potential to image patients during the early dynamic phases becomes more available in veterinary medicine (8, 9). Heterogeneous patterns of the spleen in early contrast-enhanced CT series vary considerably among subjects of the same species as well as between dogs and cats. In particular, when compared to dogs, cats may show a various distribution of splenic parenchymal enhancement on contrast-enhanced CT that is serpentine, cordlike, and archiform, similar to that reported in humans (8). However, this is only known empirically and to the author’s knowledge, no evidence-based studies have been published so far.

The spleen is a complex and dynamic parenchymal organ, which combines two distinct functional and morphologic components, the white and the red pulp (3, 10, 11). Although the major characteristics of arterial circulation are the same in all mammalian spleens, there are two distinct forms of venous circulation, those with (sinusal type) and without (nonsinusal type) venous sinuses in the reticular meshwork (12). In dogs with sinusal spleen, both open and closed circulations have been reported especially with clear evidence of an abundant closed circulation system, which was less abundant in humans than in dogs (13, 14). In cats, however, closed pathways with direct connections between vessels have not been found, with the circulation appearing to be exclusively open in nonsinusal spleens (13, 15).

In the previous study for determining the prevalence and type of splenic disease in cats, primary or metastatic neoplasia accounted for 37% of splenic lesions (16, 17). Splenic lesions are characterized by diffuse uniform infiltration or focal nodules and masses, and in contrast to dogs, focal or multifocal nodules and masses are not common in cats (18). In the recent study, round cell tumor (e.g., mast cell tumor, lymphoma), and myeloproliferative disease were the most common disease of the feline spleen (16, 19). Although diffuse splenic enlargement is particularly common in cats with round cell neoplasia and can be a useful parameter for the diagnosis, it is not diagnostically specific in that the spleen can be also enlarged in reactive processes. Furthermore, the absence of splenomegaly is not sufficient to exclude a diagnosis of infiltrative tumors, which can manifest as a normally appearing spleen with microscopic involvement only (18, 20). In humans, it is reported that in conjunction with subjective splenomegaly, the obliteration of normal heterogeneous enhancement patterns of the spleen on CT can improve the diagnostic performance for diffuse infiltrative splenic lymphoma (21). However, to the best of our knowledge, there is no study related to these changes of splenic enhancement on CT in cats with diffuse infiltrative splenic lesions (DISL).

The objective of this study was to describe the CT appearances and duration of heterogeneous splenic enhancement in clinically healthy cats and to identify certain variables influencing the duration and degree of splenic heterogeneity. We also attempted to compare those splenic enhancements with DISL. We hypothesized that (a) in most healthy cats, the spleen will exhibit a heterogeneous enhancement on contrast-enhanced CT, some of which will even be seen in the generally scanned delayed phases based on anatomical structure of the spleen; (b) DISL of cats may show homogeneous splenic enhancement in post-contrast CT scans because it interrupts variable blood flow rates as in humans.

## 2 Materials and methods

The study consisted of two separate parts. The first part of this study was a prospective analytical study and comprised exploratory experiments to describe the CT appearances and duration of splenic heterogeneity in clinically healthy cats, and to identify certain variables influencing the duration and degree of splenic heterogeneity. The second part of the study was a retrospective, case series study in which CT images of cats with DISL were retrospectively evaluated to identify the splenic heterogeneity.

### 2.1 Part 1

#### 2.1.1 Animals

A sample of 25 cats volunteered for the study by owners, students, and staff of Chungbuk National Veterinary Teaching Hospital enrolled for the study in part 1. The study protocol was approved by the Chungbuk National University Institutional Animal Care and Use Committee (CBNUA-1969-22-01), and all protocols followed the Chungbuk National University Guidelines for Animal Experiments. Written informed consent was obtained from all cat owners before study enrollment. All cats were healthy based on history, general physical examination, complete blood count, serum biochemical analysis, thoracic and abdominal radiography, echocardiography, and abdominal ultrasonography results. None of the cats had splenic lesions including splenic nodules or masses, dystrophic mineralization, or honeycomb patterns. The cats were normal, except for some with mild lipiduria or gingivitis.

#### 2.1.2 CT protocol

All CT examinations were performed on a 16-slice helical multidetector CT scanner (Revolution CT; GE Healthcare, Milwaukee, WI, United States) with the following settings: 16 rows × 0.5-mm, the helical pitch of 1.375, the helical thickness of 2.5 mm, detector coverage of 20.0 mm, rotation time of 0.98 s, a field of view of 250, tube voltage of 100 kV, and effective tube current of 40–50 mA. All cats were sedated with intravenous injections of butorphanol (0.2 mg/kg; Butophan; Myung Moon Pharm., Hwaseong, Korea), followed by anesthesia induction with propofol (6 mg/kg IV; Provive injection 1%; Myung Moon Pharm., Hwaseong, Korea). After the endotracheal tube was placed, anesthesia was maintained with 1.0–2.0% isoflurane (Terrell; Piramal Critical Care Inc., Bethlehem, PA, United States) in oxygen (1.0–1.5 L/min). All cats were positioned in sternal recumbency and short-term apnea was performed with manual hyperventilation before each scan. All cats received 2 mL/kg iohexol (Omnipaque, 350 mgI/mL; GE Healthcare Inc., Cork, Ireland) injected intravenously through a 24-gage catheter (BD Angiocath Plus; Becton Dickinson Medical Pte Ltd., Singapore) using a power injector (OptiOne; Quantum Hunex Co. Ltd., Seoul, Korea) with the rate of 2.5 mL/s. Focusing on the spleen, the scan range was equally fixed for all cats so that the total exposure time could be 10 s in every CT scan, which was 250 mm when set to the helical thickness of 2.5 mm. After obtaining unenhanced scans of the spleen, contrast-enhanced scans were performed at 10, 25, and 45 s, and then every 40 s thereafter until 245 s had past from the initiation of iohexol injection. A total of eight contrast-enhanced CT scans were obtained for each cat. If splenic heterogeneity was still present on the final scan taken after 245 s, additional scans were performed until the heterogeneity was resolved. In such cases, the time of resolution was estimated to be the time when the difference between the mean HU values of the hyperenhancing and hypoenhancing regions was measured to be 20 or less. For example, for cats that took additional scans at random time intervals after the last 245 s, if the difference between the two regions was measured to be 35 HU at 287 s and 20 HU or less at 367 s, the estimated time for resolution was determined to be 367 s.

#### 2.1.3 Image analysis

**Figure 1:**
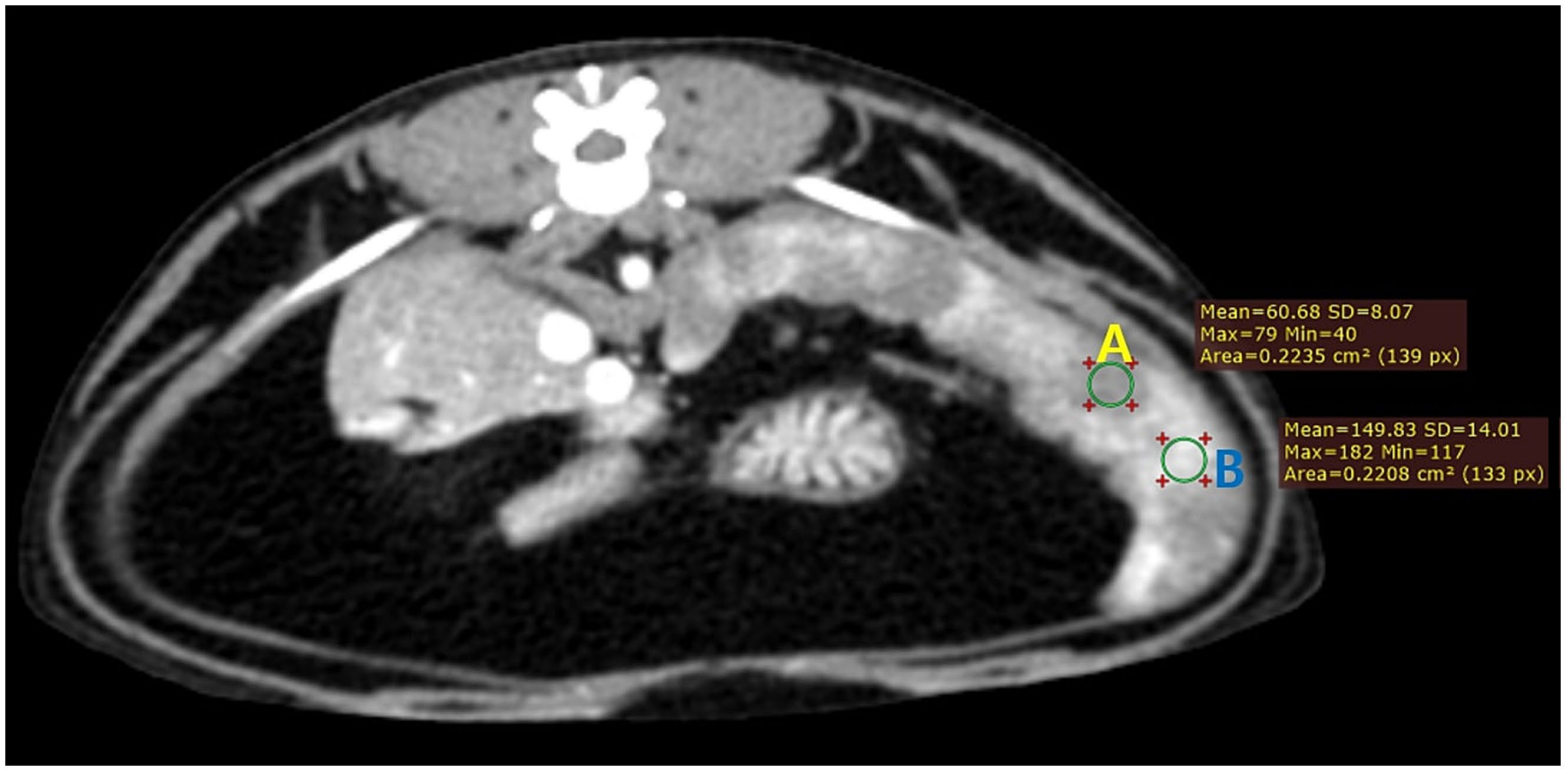
Transverse CT image of the spleen of a healthy cat (case 1) taken 25 s after the injection. Note the clear difference in the degree of contrast enhancement between different splenic areas (**A**, hypoenhancing region; **B**, hyperenhancing region). The image was reconstructed with a soft tissue algorithm and viewed with a window width of 450 and a window level of 60.

All digital images were assessed using the Digital Imaging and Communications in Medicine (DICOM) viewer (RadiAnt DICOM Viewer software; Medixant Co., Poznan, Poland) and were reviewed using a soft tissue algorithm with a window width of 450 and window level of 60. Using the clear difference in enhancement between different splenic areas in the images taken 25 s after the injection, the same area of the region of interest (ROI) cursors were placed over the corresponding hyperenhancing and hypoenhancing regions in all cats (Figure 1). After measuring the mean attenuation values (Hounsfield units, HU) of the respective ROI, its contour was imported into the corresponding images of pre-contrast and multiple post-contrast scans. The spleen was considered homogeneous if the difference between the mean HU values of the two regions was 20 or less. The degree of splenic heterogeneity was defined as a subtraction between two mean HU values of hyperenhancing and hypoenhancing areas in the images. The splenic volume was measured by summing regions of interest drawn over the spleen by tracing each slice of transverse images and by multiplying the helical thickness by the sum.

The CT images were evaluated in a standardized manner by the same author (YL) for the presence of transient splenic heterogeneity. The time between contrast media injection and the maximum visualization and resolution of splenic heterogeneity were recorded. The relationships of certain variables including age, weight, systolic blood pressure just before injecting contrast media, and splenic volume to the duration and degree of splenic heterogeneity were investigated.

### 2.2 Part 2

#### 2.2.1 Animals

Electronic medical records between January 1, 2012, and June 30, 2022, were searched to collect cases of cats with a confirmed cytological or histological diagnosis of diffuse infiltrative splenic lesions that had undergone contrast-enhanced CT at four animal medical centers including the Veterinary Teaching Hospital of Chungbuk National University (CBNU VTH), Cheongju Korea Animal Medical Center (KAMC), Daegu Animal Medical Center (DAMC), and Seoul Lucid Animal Medical Center (LAMC). Inclusion criteria required that cats had received an abdominal CT including the whole spleen and were cytologically or histologically diagnosed with diffuse infiltrative splenic lesions such as splenic lymphoma or mast cell tumor. Information retrieved from the medical files included signalment, presenting clinical signs, and final diagnosis. Cats with single or multiple nodular types of splenic involvement including hemangioma, abscess, cystic lesions, and other focal splenic lesions were excluded. Cats were also excluded if the patient’s information was incomplete.

#### 2.2.2 Image analysis

Computed tomography images were reviewed using the DICOM viewer (RadiAnt DICOM Viewer software) and using a soft tissue algorithm with a window width of 450 and window level of 60. The CT images were evaluated independently and in random order by two observers (MO and JB), each with 2 years of diagnostic imaging expertise. They were aware that the study population may include infiltrative splenic lesions but were blinded to the patient’s clinical or laboratory information. The observers were asked to determine the grade of heterogeneity of the spleens in post-contrast images. The grade of heterogeneity was estimated as follows: grade 1, remarkably heterogeneous; grade 2, moderately heterogeneous; grade 3, mildly heterogeneous; and grade 4, entirely homogeneous. To analyze the agreement of the enhancement pattern of spleen between two observers, kappa coefficient values were calculated. The measurement of splenic volume was performed in the same manner as in part 1. Splenomegaly was subjectively evaluated by observers.

#### 2.2.3 Statistical analysis

All statistical analyses and statistical figures were acquired using the statistical software SPSS 21.0 (IBMw SPSS Inc., Chicago, IL, United States) and Prism 9.0 (GraphPad Software Inc., San Diego, CA, United States). The measured data were checked for normal distribution using the Shapiro–Wilk test with the SPSS software. Correlation and regression analyses were performed for the duration and the degree of splenic enhancement, and variables including age, weight, systolic blood pressure, and splenic volume. If the results were normally distributed, the Pearson correlation coefficients were analyzed, and if the results were non-normally distributed, the Spearman’s rank correlation coefficients were analyzed. Agreement between the observer’s grading of the splenic homogeneity was measured using the kappa statistics. The kappa coefficient values were evaluated as follows: <0.20, poor agreement; 0.21–0.40, fair agreement; 0.41–0.60, moderate agreement; 0.61–0.80, substantial agreement; and 0.81–1.00, almost perfect agreement. Statistical significance was set at *p* < 0.05.

## 3 Results

### 3.1 Part 1

Fourteen cats met the inclusion criteria and were enrolled in the prospective part of the study; six males and eight females with a mean age (±SD) of 5.1 (±2.7) years (range, 1.2–10.3 years) and the mean body weight (±SD) of 5.0 (±1.1) kg (range, 4.0–7.5 kg) at the time of enrollment. Only one female cat (*n* = 1, 7%) in the study was intact, and the remaining cats were neutered/spayed. Represented breeds included domestic shorthair (*n* = 8), mixed breed (*n* = 3), and one each of Scottish Straight, Siamese, and Russian Blue. The median splenic volume and systolic blood pressure were 33.1 cm^3^ (range, 24.8–52.9 cm^3^) and 74.5 mmHg (range, 63–92 mmHg), respectively.

**Figure 2:**
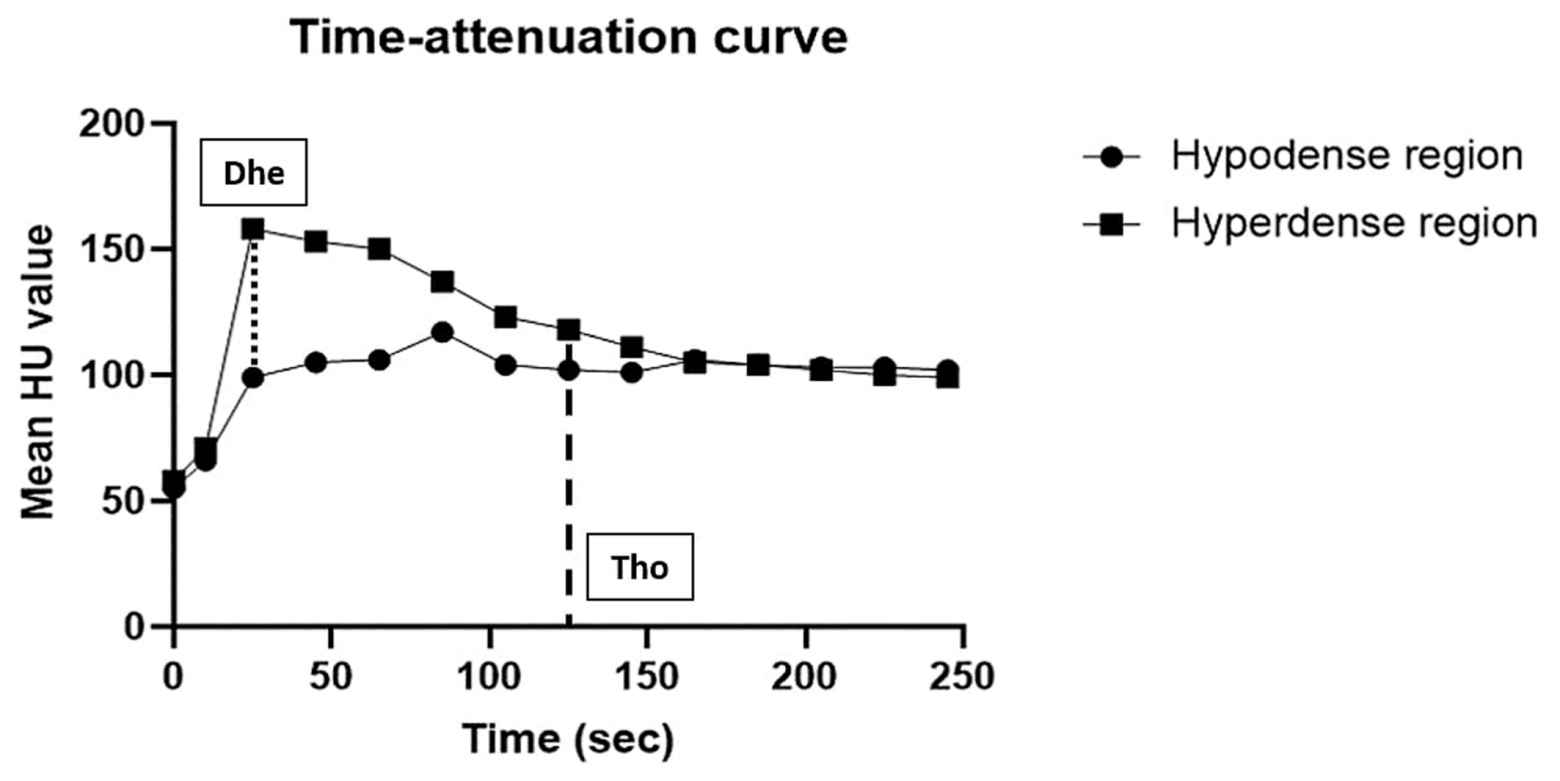
Example of a time-attenuation curve of two differently enhanced regions (hypoenhancing and hyperenhancing regions) in every post-contrast dynamic phase of the spleen in a healthy cat (case 2). The *x*-axis represents time (s) and the *y*-axis represents the mean HU value. The degree of splenic heterogeneity (Dhe) was defined as a subtraction between two mean HU values of hyperenhancing and hypoenhancing areas in the images. The spleen was considered homogeneous if the difference between the mean HU values of the two regions was 20 or less, and the time taken to reach homogeneity (Tho) was measured.

**Figure 3:**
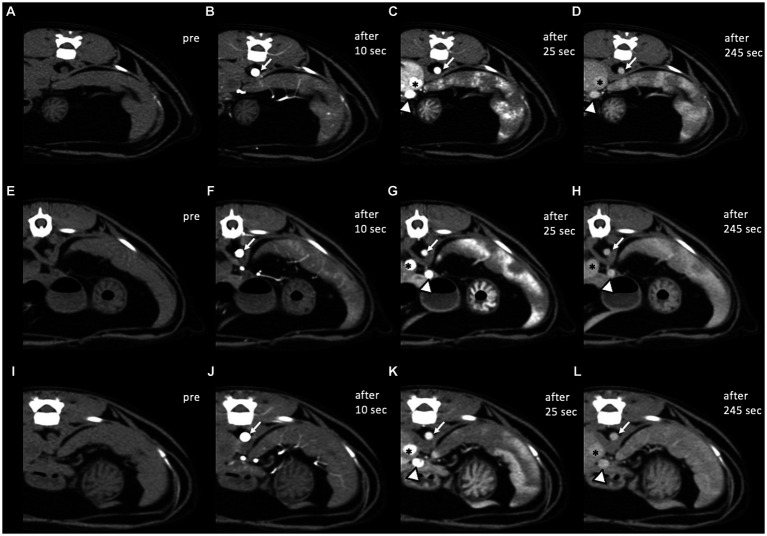
Pre- and post-contrast CT transverse images in a soft tissue window of three healthy cats. **(A–D)** Case 3, **(E–H)** case 6, and **(I–L)** case 7. In images taken 10 s after the injection **(B,F,J)**, only the abdominal arteries including aorta (white arrows) were enhanced with the peak enhancement. While in images taken 25 s **(C,G,K)** and 245 s **(D,H,L)** after the injection, caudal vena cava (asterisks) and portal vein (arrowheads) were also enhanced, and especially after 25 s, the peak enhancement of the veins was confirmed. Compared to case 7, heterogeneous splenic enhancement still existed in the last delayed phase in cases 3 and 6. CT images were obtained with a slice thickness of 2.5 mm, reconstructed with a soft tissue algorithm, and viewed with a window width of 450 and window level of 60 **(A–L)**. CT images were acquired with a kVp of 100 and 40–50 mA.

In all cats, standardized time-attenuation curves of two differently enhanced areas of the spleen were created from selected ROIs, representing the mean attenuation value (HU) in relation to time (s) (Figure 2). In images taken 10 s after the injection, only the abdominal arteries including aorta were enhanced with the peak enhancement in all cats. While in images taken at and after 25 s, caudal vena cava and portal vein were also enhanced, and especially at 25 s, the peak enhancement of the veins was confirmed (Figure 3). Heterogeneous splenic enhancement was seen in all cats (14/14, 100%). In all cats, the maximum degree of splenic heterogeneity was observed at 25 s after the injection of contrast media with the median degree of splenic heterogeneity being 109 HU (range, 59–198 HU) (Figure 3), and the median of mean HU values of the hyperenhancing and hypoenhancing area of the spleen being 178 HU (range, 130–259 HU) and 66.5 HU (range, 59–99 HU), respectively. The median degree of splenic heterogeneity in 10 and 45 s, and the last delayed phase taken 245 s after the injection was 4 HU (range, 0–31 HU), 92.5 HU (range 47–177 HU), and 44.5 HU (range 0–92 HU), respectively. The median time taken to reach splenic homogeneity after injecting contrast media was 329.0 s (range, 125–604 s). Based on the estimated time for resolution of splenic heterogeneity calculated for cats scanned additionally after the last delayed phase, splenic heterogeneity was still persistent at 5 min in nine of 14 cats (64.3%), at 6 min in five cats (35.7%), at 7 min in four cats (28.6%), at 8 min in two cats (14.3%), and at 10 min in one cat (7.1%).

No statistically significant correlations were seen between the duration of splenic heterogeneity and variables including weight (*p* = 0.06), age (*p* = 0.94), systolic blood pressure (*p* = 0.22), and splenic volume (*p* = 0.81). In addition, no statistically significant correlations were seen between the degree of splenic heterogeneity in the images taken 25 s after the injection and variables including weight (*p* = 0.11), age (*p* = 0.68), systolic blood pressure (*p* = 0.07), and splenic volume (*p* = 0.34).

### 3.2 Part 2

#### 3.2.1 Clinical findings

A total number of five cats were included in the retrospective portion of this study; three males and two females with a mean age (±SD) of 7.4 (±3.4) years (range, 2.0–11.3 years) and a mean body weight (±SD) of 5.1 (±1.1) kg (range, 4.0–6.4 kg) at the time of the CT scan. All cats were neutered or spayed. The sample included domestic shorthair (*n* = 3), Siamese (*n* = 1), and Scottish Fold (*n* = 1). DISL diagnoses included lymphoma (*n* = 3, 60%) and mast cell tumor (*n* = 2, 40%). Final diagnoses of the splenic lesions were performed by cytology in 3/5 (60%) cases including two lymphomas and one mast cell tumor, and by surgical excision/biopsy in 2/5 (40%) cases including lymphoma and mast cell tumor (Table 1).

**Table 1:** Individual characteristics, the time interval between pre- and post-contrast scans, and the grade of subjective splenic heterogeneity by two observers of cats with DISL.

Patient no.	Age	Breed	Sex	BW (kg)	Splenic volume (cm^3^)	Final diagnosis	Time interval (s)	Subjective heterogeneity grade
1	2 years	Scottish	CM	4.0	107.5	Lymphoma	45	4/4
2	7 years 4 months	DSH	CM	6.2	177.9	MCT	60	4/4
3	11 years 4 months	Siamese	CM	6.4	104.3	Lymphoma	74	2/3
4	7 years 5 months	DSH	SF	4.3	280.2	MCT	120	3/3
5	9 years	DSH	SF	4.6	201.4	Lymphoma	10, 25, 120	4/4

DISL, Diffuse infiltrative splenic lesions; BW, Body weight; s, seconds; CM, Castrated male; DSH, Domestic shorthair; MCT, Mast cell tumor; and SF, Spayed female.

#### 3.2.2 CT acquisition technical parameters

Computed tomography examinations were performed using four different scanners: CBNU VTH, 4-slice helical CT scanner (Hi-Speed QX/I; GE Healthcare, Milwaukee, WI, United States); KAMC, 16-slice helical CT scanner (SOMATOM Scope; SIEMENS, Tokyo, Japan); DAMC, 16-slice helical CT scanner (Light Speed; GE Healthcare, Milwaukee, WI, United States); and LAMC, 64-slice helical CT scanner (Light Speed VCT; GE Healthcare, Milwaukee, WI, United States). The imaging parameters of the four CT scanners were as follows: slice thickness of 1.25–1.5 mm, the helical pitch of 0.75–1.5, tube voltage of 120–130 kV, effective tube current of 100–125 mA, a field of view of 250–500 mm, and matrix size of 512 × 512. All scans were performed under general anesthesia and positioned in sternal recumbency, with short-term apnea performed using manual hyperventilation before each scan. All cats received an intravenous injection of the nonionic contrast media, iohexol (Omnipaque, 350 mgI/mL), at a dose of 2.0–3.0 mL/kg, and contrast media were injected either manually or with a power injector at the rate of 1.5–2.5 mL/s. All cats underwent contrast-enhanced CT. In four cases, a single post-contrast scan was acquired at 45 s (patient 1), 60 s (patient 2), 74 s (patient 3), and 120 s (patient 4), and in one case, a tri-phasic protocol using bolus tracking method was performed (patient 5). The ROI for the bolus tracking was placed in the aorta between carina and diaphragm. Scan monitoring for the aorta was started concurrently with the administration of the contrast medium. The first scan started 10 s after contrast administration, and following scans were performed with a delay of 15 and 110 s after the first scan.

#### 3.2.3 CT characteristics of DISL cats

**Figure 4:**
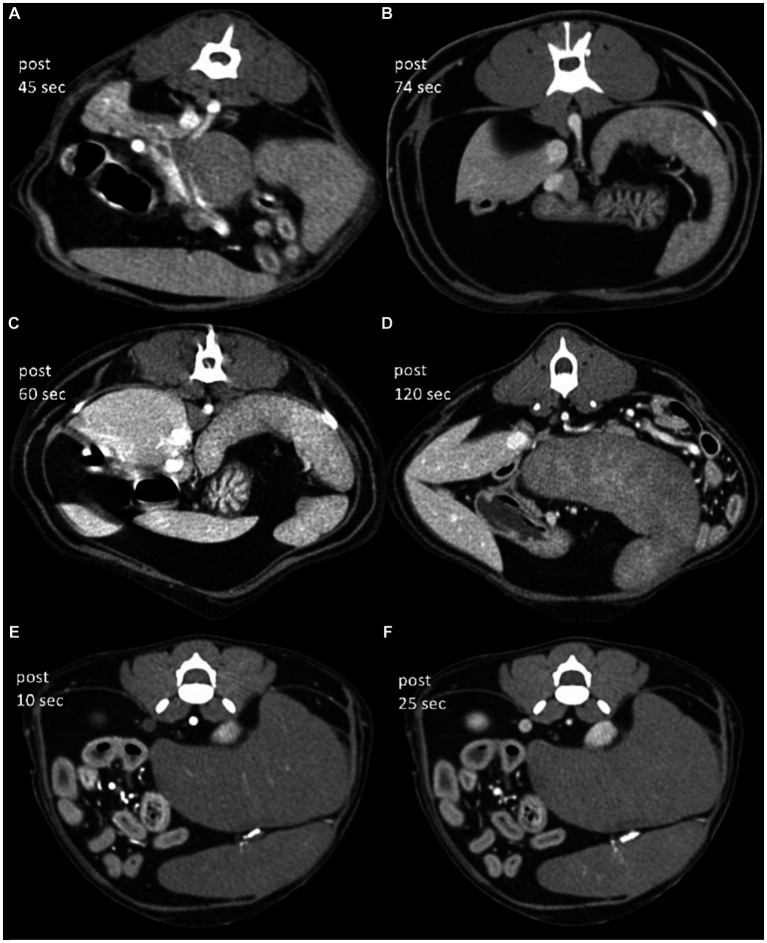
Post-contrast CT transverse images in a soft tissue window of cats with diffuse infiltrative splenic lesions. **(A)** Lymphoma (patient 1), **(B)** lymphoma (patient 2), **(C)** mast cell tumor (patient 3), **(D)** mast cell tumor (patient 4), and **(E,F)** lymphoma (patient 5). Images were obtained with a slice thickness of: 1.3 mm **(A,C–F)** and 1.5 mm **(B)**, reconstructed with a soft tissue algorithm and viewed with a window width of 450 and window level of 60 **(A–E)**. The approximate time for image acquisition after intravenous contrast administration was variable for each patient, which took 45 s (patient 1), 120 s (patient 4), 74 s (patient 2), 60 s (patient 3), and 10/25 s (patient 5) after intravenous contrast administration, respectively.

The median splenic volume was 178.0 cm^3^ (range, 104.3–280.2 cm^3^). The subjective heterogeneity of spleen of two observers in five patients were rated as “grade 2” (1/10; 10%), “grade 3” (3/10; 30%), and “grade 4” (6/10; 60%) (Table 1; Figure 4). The kappa statistic showed an overall substantial agreement (kappa = 0.64) between observers (*p* = 0.04).

## 4 Discussion

In our study, heterogeneous enhancement of spleens was observed in all 14 healthy cats. Similar heterogeneous splenic enhancement in cats have been reported in contrast-enhanced ultrasound (22), however, to the author’s knowledge, no evidence-based studies with multiple series of contrast-enhanced CT beyond the equilibrium phase have been published so far. In contrast-enhanced ultrasound, the time taken for the resolution of splenic heterogeneity after the ultrasound contrast agent injection was about 30 s (22). However, splenic heterogeneity in contrast-enhanced CT lasted more than 5 min in nine of 14 cats (64.3%) in this study. Also, maximum heterogeneity of the spleen was observed in the images taken 25 s after the injection in all healthy cats in the present study, not in the images taken 10 s after with peak aortic enhancement. These findings were somewhat different from the previously known fact that heterogeneous enhancement of the splenic parenchyma usually appears in the arterial phase, and in the delayed phase, the normal spleen parenchyma shows homogeneous enhancement (8, 9).

There are two distinct forms of venous circulation in mammalian spleens, those with (sinusal type) and without (nonsinusal type) venous sinuses in the reticular meshwork (12). The sinusal spleen comprises humans, dogs, and rats; cats and mice have nonsinusal spleen. Compare to sinusal spleens with venous sinuses, which are larger in size with greater abundance and richly anastomosing plexus, nonsinusal spleens have pulp venules, which are shorter and smaller in caliber, and nonanastomosing (10). Therefore, regarding the blood entry system into the venous circulation, sinusal spleens like in dogs show rapid flow from the arterial capillaries to veins (13, 14). In cats, however, closed pathways with direct connections between vessels have not been found, with the circulation appearing to be exclusively open in nonsinusal spleens (15).

Anecdotally, compared to cats, the authors have seen many cases of splenic heterogeneity during arterial phase in dogs, which in turn showed homogeneous enhancement in venous or early delayed phases. Because of the anatomical difference in the circulatory pathways between species, we suspect that the distinct and persistent heterogeneity of cats’ spleens compared to dogs may be due to their unique nonsinusoidal vascular structure, in which the circulation appears to be exclusively by slow flow pathway. Thus, care should be taken when interpreting splenic lesions even in the generally scanned delayed venous phase since these normal heterogeneous enhancements may mask and mimic actual lesions.

In human medicine, it is suggested that the frequency of transient splenic heterogeneity in dynamic CT scans is influenced by several factors, including the injection rate of contrast media, presence or absence of splenomegaly, age, and portal venous pressure (1, 2, 4). In comparison, all 14 healthy cats in this study showed transient splenic heterogeneity, regardless of variables including body weight, age, systolic blood pressure, or splenic volume. In addition, the degree of splenic heterogeneity in the images taken 25 s after the injection did not significantly correlate with the variables. However, a small population may have prevented the achievement of significance from the data.

In this study, the same protocol of anesthesia using butorphanol, propofol, and isoflurane was performed in all cats. It is well known that a variety of anesthetic agents dilate the spleen and lower systemic blood pressure, both of which can reduce splenic blood flow. Also, as in the study looking at the effects of anesthesia in contrast-enhanced ultrasound (22), it is likely that anesthesia-induced splenic congestion amplifies the heterogeneous enhancements in cats during CT scans. Accordingly, the perfusion of the spleen may vary depending on which anesthetic agents are used, and when CT scans are performed without anesthesia such as using the device, which immobilizes cats the duration of the heterogeneous splenic enhancement may be shorter than in this study. Therefore, additional studies will be needed in the future on the duration of the splenic heterogeneity according to the type of anesthetics and comparison with awake cats.

In the retrospective part of this study, along with diffuse splenomegaly, the spleen was mainly homogeneous in post-contrast CT scans in the DISL group. In humans, it has been suggested that infiltrative splenic lymphoma typically affects the periarterial lymphatic sheaths (PALS) of the white pulp. As a result, it is believed that the white pulp’s involvement, which reduces its flow rate, is primarily responsible for the obliteration of splenic heterogeneity (21). Therefore, it is likely that a similar mechanism suggested in humans led to the observation of these findings in cats with DISL; however, more comprehensive research including histological evaluations is necessary. Compared to dogs, cats are more likely to develop infiltrative diseases of the spleen, with round cell tumors including mast cell tumors and lymphoma being the most common tumors (16, 18). While diffuse splenomegaly is frequently observed in cats with round cell tumors and can help with diagnosis, the spleen can also enlarge as a result of reactive processes. Moreover, infiltrative tumors can be diagnosed even in the absence of splenomegaly since they can present as a normally looking spleen with microscopic involvement only (18, 20). Therefore, along with diffuse splenomegaly, homogeneous enhancement of the spleen in post-contrast CT scans may indicate malignant infiltrative lesions in cats as shown in humans with infiltrative splenic lymphoma. However, other benign infiltrative lesions such as extramedullary hematopoiesis and lymphoid hyperplasia, which could also result in diffuse splenomegaly, were not included in this study, and therefore, the distinction between malignant and benign infiltration could not be identified. Further studies including a large cohort of cats with various DISLs are required to find differences in splenic enhancements between malignant and benign infiltrations.

Because of its retrospective design in the second part of the study, protocols for contrast-enhanced CT in each hospital differed in terms of scan condition, technique, and resolution. Also, only one patient had a tri-phasic CT scan performed, while the others had single post-contrast studies. Therefore, there was a limitation in that it was difficult to accurately determine whether homogeneity was confirmed from the beginning of the contrast administration or whether the heterogeneity was lost afterward. However, in the prospective part of the study, the median time taken to reach splenic homogeneity after injecting contrast media was 329 s and even the minimum time was 125 s, and all patients in the retrospective part were scanned earlier than these times in post-contrast imaging. Therefore, it can be hypothesized that the normal heterogeneous splenic enhancement that appeared in healthy cats were not visible or appeared much weaker in the patients with DISL that did not proceed with the tri-phasic CT scan.

Our study had several limitations. First, in the prospective part of the study, detailed statistical analysis and the achievement of significance from the data were prevented by the limited population. Second, the spleens were presumed to be normal without performing either cytologic or histologic examinations. All cats were however deemed healthy except for some with mild lipiduria or gingivitis, based on thorough examinations including physical examination, hematologic analysis, thoracic and abdominal radiography, echocardiography, and abdominal ultrasonography. Third, the bolus tracking method was not used in this study to compare the images taken at the same time in all cats. Therefore, there is a limit to whether the specific time taken corresponds exactly to the arterial, venous, and delayed phases of each cat. Lastly, we only examined a small number of patients with DISL, which might have selection bias and could cause the results to be overestimated.

In conclusion, the findings support that transient splenic heterogeneity is highly common in cats undergoing contrast-enhanced CT and can be seen even in the generally scanned delayed phases, which can help with the interpretation of CT images of feline spleens. In addition, our results suggest that homogeneous splenic enhancement along with splenomegaly on CT images could be useful as a diagnostic indicator of DISL in cats. Further studies with more comprehensive research including the histological evaluation and a large cohort of cats with various DISLs are needed.

## Data availability statement

The original contributions presented in the study are included in the article/supplementary material; further inquiries can be directed to the corresponding author.

## Ethics statement

The animal studies were approved by Chungbuk National University Institutional Animal Care and Use Committee (CBNUA-1969-22-01). The studies were conducted in accordance with the local legislation and institutional requirements. Written informed consent was obtained from the owners for the participation of their animals in this study.

## Author contributions

YL: Conceptualization, Data curation, Formal Analysis, Investigation, Methodology, Project administration, Resources, Software, Supervision, Validation, Visualization, Writing – original draft, Writing – review & editing. SK: Writing – review & editing. MO: Writing – review & editing. JB: Writing – review & editing. ML: Writing – review & editing. JC: Writing – review & editing. JA: Writing – review & editing. TA: Writing – review & editing. DC: Conceptualization, Data curation, Writing – review & editing.
